# Scale-up of Aflatoxin Purification by Centrifugal Partition Chromatography

**DOI:** 10.3390/toxins15030178

**Published:** 2023-02-25

**Authors:** Gábor Endre, Babett Edit Nagy, Dániel Hercegfalvi, Csenge Kasuba, Csaba Vágvölgyi, András Szekeres

**Affiliations:** Department of Microbiology, Faculty of Science and Informatics, University of Szeged, Közép fasor 52, H-6726 Szeged, Hungary

**Keywords:** large scale, mycotoxin purification, liquid–liquid chromatography, counter-current chromatography

## Abstract

Aflatoxins (AFs) are a group of secondary metabolites that cause several diseases in both animals and humans. Since the discovery of this group of toxins, several effects were revealed, such as hepatic changes, carcinoma, failure, and cancer of the liver. In the European Union, there are concentration limits for this group of mycotoxins in food and feed products; thus, these substances are required in their pure forms to prepare reference standards or certified reference materials. In our present work, a liquid–liquid chromatographic method utilizing a toluene/acetic acid/water ternary system was improved. In order to enhance the purification and gain a higher amount of pure AFs in one separation run, a scale-up of the previous separation was carried out. In several scale-up steps—including the determination of the maximum concentration and volume to load on a 250 mL rotor via a loop and via a pump as well, and the quadruplication of the entire separation procedure to a 1000 mL rotor—an efficient scale-up was achieved. Utilizing a 250 mL rotor in an 8-hour workday, altogether approximately 2.2 g of total AFs could be purified with 8.2 liters of solvent, while on a 1000 mL column, approximately 7.8 g AFs could be prepared, utilizing around 31 liters of solvents.

## 1. Introduction

Aflatoxins (AFs) are a group of mycotoxins discovered in the 1960s. This group of substances were firstly described in connection with the loss of several thousand turkeys and poultry [1,2]. It was shown that these toxins cause hepatic change in poultry and carcinoma of the liver in rats [3]. When food contaminated with AFs is consumed, it can cause a series of health issues in humans, including acute hepatic necrosis, acute liver failure, and liver cancer [4].

AFs are produced mainly by two *Aspergillus* species called *Aspergillus flavus* and *A. parasiticus*, belonging to the section *Flavi* of the *Aspergillus* genus [5,6]. Recently, 18 known analogs of aflatoxins were described, and among them, three series have significant importance from the food safety point of view, including the B-, G-, and M-series [6]. The B-series (AFB_1_ and AFB_2_) and the G-series (AFG_1_ and AFG_2_) were produced mainly by fungi, a group of structurally related difuranocoumarins, while the M-series (AFM_1_ and AFM_2_) could be detected primarily in animal tissues and fluids (milk and urine) as the hydroxylated metabolic products of the fungal AFs [7]. Among these compounds, AFB_1_ was shown to be the most toxic, with carcinogenic, mutagenic, and teratogenic properties leading to classify it as a Group 1 carcinogen by the IARC (1993) [8]. 

There have been several methods developed for the preparative separation and preparation of AFs, including the normal-phase (alumina and silica gel) [1,6,7,9,10] and the reversed-phase preparative high-performance liquid chromatography [11,12], as well as recently the centrifugal partition chromatography (CPC), which is a hydrostatic type of liquid–liquid chromatography [13]. Normal-phase chromatography is the oldest preparative chromatographic technique, which was first developed for the separation of the aforementioned toxins [1,6,7,10]. On alumina, the two groups of AFs (AFBs and AFGs) were separated by Nesbitt et al. [6], while when using silica gel, the four main AFs were successfully prepared with purities above 90% [7]. High-purity AFs were also achieved by several consecutive purifications with different elution ratios by Stubblefield et al. and De Jesus et al [9,10]. In 1977, preparative HPLC was used to have AFM_1_, AFB_1_, and AFG_1_ purified, from the mixtures of AFM_1_–M_2_, AFB_1_–B_2_, and AFG_1_–G_2_ [11]. At the end of the separation procedure, 80 mg of AFB_1_ and AFG_1_ and 9–10 mg of AFM_1_ could be obtained, including at least two reverse-phase chromatographic runs for each compound. From 100 g of a contaminated and grained peanut sample, 500 µL of cleaned-up AF extract was injected to a preparative HPLC column [12]. It was demonstrated that even though performing multiple clean-up steps, the separation of these molecules turned out difficult, although the four AFs were separated from each other and from the impurities. Furthermore, in our previous paper, the successful separation and purification of AFs was described via CPC, achieving remarkable high yields and excellent recovery. In our previous experiment, a total of 1350 mg AFs was purified from 1.5 g crude *A. parasiticus* extract, applying only a single liquid–liquid chromatographic run [13].

Scaling up in preparative chromatography can be carried out by applying different models of the calculations, starting from the existing basic separations [14,15]. In the case of liquid–liquid chromatography, especially for centrifugal partition chromatography (CPC), a specific scale-up procedure termed the “linear scale up method” can be utilized [16,17]. In this chromatographic technique, the volume of the column is increased to achieve a larger yield, which is applied as the linear transfer factor to the proportionate alteration of other separation parameters. Another scaling-up approach is the “free space between peaks” method, which is an experimental method also adaptable for the maximum load per run or for maximum productivity [18].

In the present study, the successful scale-up of our previous CPC process was achieved, optimizing the loading capacity of the method and applying a four times higher volumetric liquid–liquid chromatographic column. 

## 2. Results 

### 2.1. Determining the Maximum Loading Capacity on a 250 mL Rotor

According to our optimized method, the four main AFs were separated and purified by CPC in the ascendant mode on a 250 mL rotor, applying the upper and lower phase of the toluene/acetic acid/water 30:24:50 (v/v/v%) ternary system as the mobile and stationary phases, respectively. In a single chromatographic run, the four main AFs (AFB_1_, AFB_2_, AFG_1_, and AFG_2_) were separated and together from an injected amount of 90 mg crude AF extract, and a total of 81 mg of AFs was purified, reaching a 97.3% average purity and a 92.1% average recovery for the AFs [13]. 

**Figure 1:**
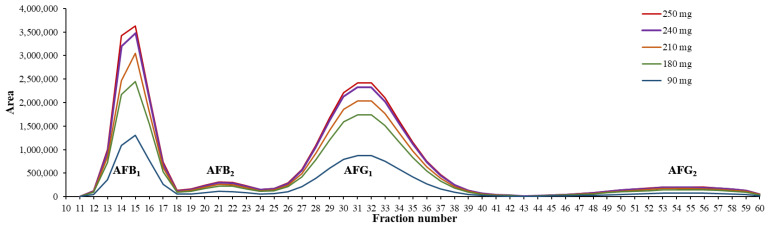
Fractograms of the CPC separations with increasing sample concentration injected with 10 mL loop on a 250 mL rotor.

According to the developed chromatographic conditions, the optimal flow rate was 15 mL/min, and the rotor speed was 1800 rpm. With these conditions, 60 mL of the stationary phase (24% of column volume) was extruded, providing this volume for the mobile phase (V_m_), and the system pressure was 46 bar. Altogether, 60 fractions were collected in a 75-minute-long separation (Figure 1). Due to the stability of the method, the whole process was fully automatized, including the column filling, equilibration, elution, and the fraction collection as well.

To improve the capacity of the separation, initially the maximum load of the 250 mL column was determined using the available 10 mL sample loop of the instrument. For this purpose, increasing amounts of the crude AF extract (90 mg, 180 mg, 210 mg, 240 mg, and 270 mg) were injected into the column to separate with the optimal conditions. In each case, the dried extracts were prepared in a 10 mL mixture of the upper and lower phases (1:1, v,v%) of the ternary system. However, while the extracts were soluble at the lower concentrations, the extract could not have been dissolved completely at the highest applied amount (270 mg). Therefore, the fine stepwise tune of the applicable extract amount was carried out in 10 mg increments within the 240 mg–270 mg range, where the maximum solubility was determined to be 250 mg of the crude AF mixture (25 mg/mL). 

Based on the resulting fractograms of each separation (Figure 1), it can be concluded that the resolution of the system remained constant, and no overlapping of the neighboring peaks was noticed despite the increasing loading concentrations. Therefore, the available highest loading capacity in the applied biphasic system with the system-provided 10 mL loop injection is a maximum of 250 mg.

**Figure 2:**
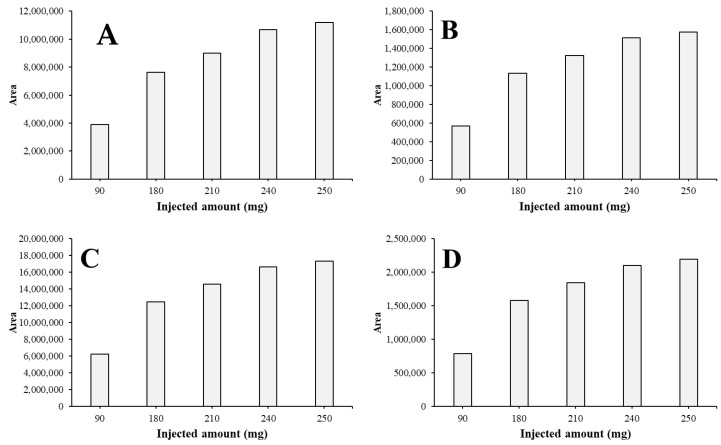
Area of collected AFB_1_ (**A**), AFB_2_ (**B**), AFG_1_ (**C**), and AFG_2_ (**D**) at different injected amounts on a 250 mL rotor.

Regarding the collected AFs of each run, it can be concluded that as the solvent system gets more saturated with the compounds, the collected amounts are bending into a saturation curve (Figure 2). This also confirms that with this injection method, the separation cannot be more enhanced in this way; the system is at the maximum capacity.

**Figure 3:**
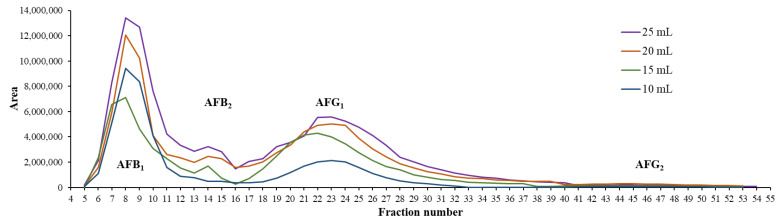
Fractogram of the CPC separations with increasing injection volumes on a 250 mL rotor.

After the maximum of the solubility was determined, the next step was to determine the maximum loading volume with the highest concentration to improve the capacity of the whole purification system. Injections in increments of 5 mL were carried out via the built-in pump of the PLC system from 10 mL until 10% of the V_c_ (25 mL), which is the usually applied value as the maximum loading volume onto a column in the case of liquid–liquid chromatography [19]. Based on these volumes, 250 mg, 375 mg, 500 mg, and 625 mg of the crude mixture was injected into the 250 mL column. According to the resulting fractograms of the CPC runs, it can be concluded that with increasing injection volumes, the resolution remained nearly the same, and the peaks did not widen dramatically (Figure 3). It can also be seen that the system was stable, and the retentions of the compounds were not shifted. These phenomena and the application of the pump injection allow both the sequential application of the CPC runs and the collections of each AF into the same tubes within the consecutive separations. This sequence of the CPC separations can be set in the control software, providing the fully automatized continuous purification of the AFs.

All fractions that contained one AF with 95% purity or more were pooled. The fractions 5–11, 13–16, 17–31, and 40–54 were combined for AFB_1_, AFB_2_, AFG_1_, and AFG_2_, respectively, during each separation.

**Figure 4:**
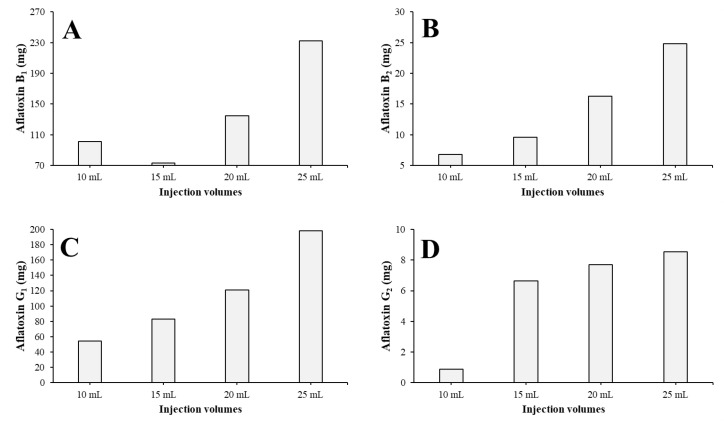
Pure (≥95%) AFB_1_ (**A**), AFB_2_ (**B**), AFG_1_ (**C**), and AFG_2_ (**D**) content of each injection on a 250 mL column.

The pure (≥95%) content was summarized for each AF in each injection (Figure 4). It can be concluded that with increasing injection volumes, the amount of pure material increases, but linearly it increases by an exponential rate, except in the case of AFG_2_ (Figure 4D). This determines that if the injection volume could be greater, the larger amount of pure AFs could be gained. Because the maximum of the injection volume on a column is 10% of the V_c_ and 25 mL of the most concentrated possible crude extract was injected to a 250 mL column, the system was utilized in the most advantageous way.

### 2.2. Linear Scale-up of the Purification to a 1000 mL Column

As the maximum loading capacity was determined on the 250 mL column including both the concentration and volume, the entire separation was scaled up to the 1000 mL rotor. During this procedure, the injected amount and the flow rate applied on the 250 mL column were quadruplicated according to the ratio of the 250 mL and 1000 mL rotor volumes [16]. To achieve the same resolution during the scale-up, the centrifugal force fields on both columns had to be the same. In the case of the 250 mL column, the rotation was 1800 rpm, creating a 435× *g* centrifugal force field, which can be reached by setting the rotation speed to 1267 rpm on the 1000 mL column.

The determination of the maximum or optimal load was carried out in the same method as it was applied on the 250 mL rotor. Injections via the pump utilizing the solution of 25 mg/mL crude extract were performed in 20 mL increments from 4% of the V_c_ (40 mL) to 10% of the V_c_ (100 mL); thus, the injected amounts of the crude AF extracts were 1 g, 1.5 g, 2 g, and 2.5 g, respectively. Furthermore, based on the criterion of the linear scale-up [16], the flow rate was quadruplicated from 15 mL/min to 60 mL/min. The extruded stationary phase was 250 ± 5 mL regarding all the injections (25% of V_c_), which is similar to the V_m_ on the smaller rotor. In each run, 90 fractions were collected. 

**Figure 5:**
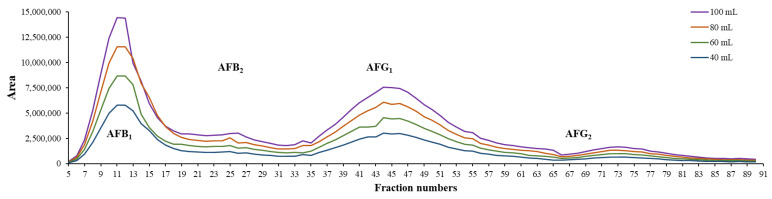
Fractogram of injections with increasing injection volumes on a 1000 mL rotor.

With the application of the described linear scale-up approach, the CPC runs could also be performed successfully on the higher-volume column, where the separation possessed similar parameters than were observed in the case of the 250 mL rotor (Figure 4). Furthermore, during the series of injections of the crude extracts in incrementing amounts, the system remained stable despite the increasing volumes of the injections (Figure 5.).

As this chromatographic technique relies on a basic liquid–liquid extraction method, it can be expected that the resolution will stagnate, and the following peaks will not overlap, because the distribution of each compound will not change despite the increasing volume or concentration. This phenomenon can be changed in one case if the phases become oversaturated with the compounds. Because the solubility was initially determined, this problem was avoided (Figure 5).

**Figure 6:**
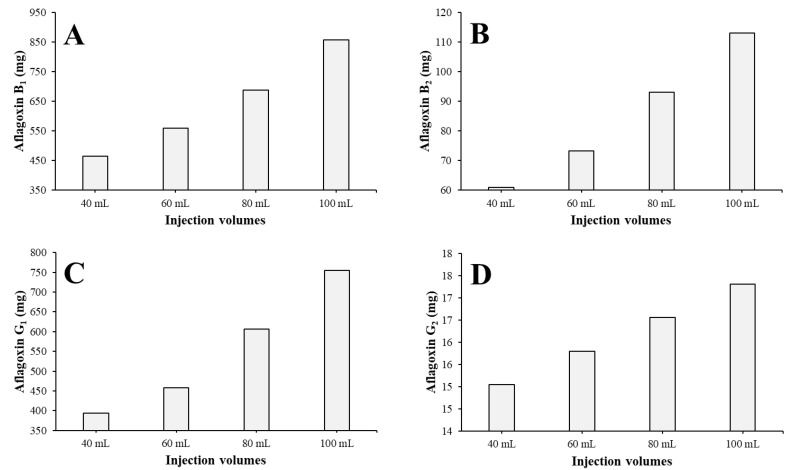
Pure (≥95%) AFB1 (**A**), AFB2 (**B**), AFG1 (**C**), and AFG2 (**D**) content of each injection on a 1000 mL column.

The fractions containing pure (≥95%) AFs were worked up and the pure AF content of each run and each compound was determined (Figure 6). The fractions 5–18, 20–32, 33–65, and 67–90 were combined for the total AFB1, AFB2, AFG1, and AFG2 workups, respectively, at the end of each run. It can be concluded that the gained pure AF amount increases linearly instead of exponentially, as was observed on the 250 mL column in all the cases (Figure 6). 

### 2.3. Product Purity

**Figure 7:**
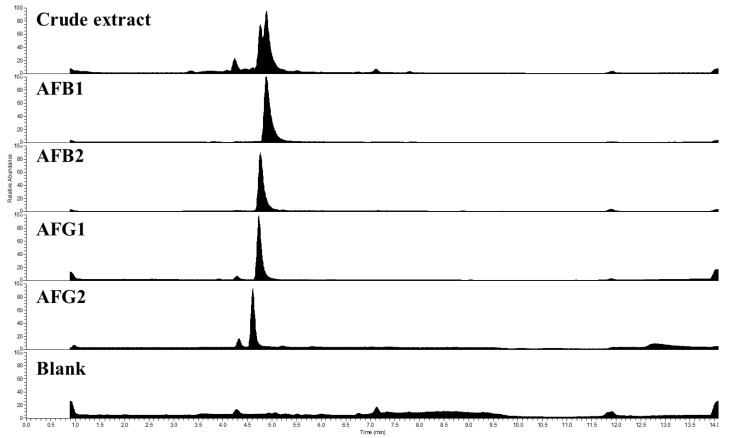
Total ion chromatograms (TIC) of the separated crude extract, the pure AFB1, AFB2, AFG1, AFG2, and a blank run.

**Figure 8:**
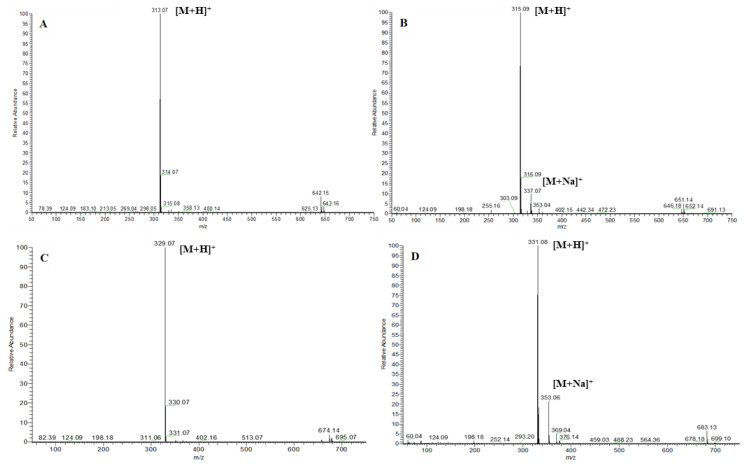
Mass spectra of the purified AFB_1_ (**A**), AFB_2_ (**B**), AFG_1_ (**C**), and AFG_2_ (**D**).

After each run, the combined pure fractions were analyzed by the HPLC-UV-HRMS technique. The purities of the resulted compounds were calculated from the HPLC-UV chromatograms and were confirmed by high-resolution mass spectrometry. The total ion chromatograms (TIC) and mass spectra of the four purified AFs are shown in Figure 7 and Figure 8, respectively.

### 2.4. Yield of the Entire Scale-up Procedure

In all the purifications, AFB_1_ resulted in a light yellow powder, while AFB_2_, AFG_1_, and AFG_2_ resulted in white powders. After the separations, the yields, purities, recoveries, and solvent consumption of each performed run were summarized (Table 1). It seems that when the crude extract was injected via the loop, the recoveries more or less stagnated while more pure material could be gained. On the other hand, changing the loop injection to the pump injection causes losses in the recovery (from 89.6% to 71.6%). It also decreased when higher amounts of crude extracts were injected onto the 1000 mL column, regarding that the aim was to gain pure (≥95%) AFs.

## 3. Discussion

According to the literature, CPC usually allows for rapid and inexpensive method development, higher throughput, higher yields, and reduced costs for the preparative purifications compared to typical preparative HPLC techniques [20]. Based on these unique advantages and the high resolution offered by CPC, this technique has been applied for the separation of certain mycotoxins [21,22,23,24,25,26]. 

In the work of Szekeres et al., fumonisin B_1_ and fumonisin B_2_/B_3_ mycotoxins were separated from the pre-purified extract of the *Fusarium verticillioides* rice culture by CPC in a 100 min run, with a purity of fumonisin B_1_ above 98% and a total recovery of 68% [21]. B-type fumonisins have also been purified from a *F. verticillioides* maize culture by two consecutive CPC separation steps. As a result, a total of 500 mg of fumonisin B_1_, 100 mg of B_2_, and 50 mg of B_3_ were obtained, with a purity above 98% from 1 kg of the maize culture; however, the recovery was not reported [22]. For the mycotoxins, nivalenol and fusarenon-X were purified by passing a crude acetonitrile extract of *F. graminearum* cultured on a pressed barley medium through silica gel and then running two consecutive CPC separations. From 1 kg of a pressed barley culture, 340 mg nivalenol and 600 mg fusarenon-X were obtained with a purity of 103% and 77%, respectively. The final recoveries of nivalenol and fusarenon-X calculated from their contents in the starting acetonitrile extract were 44 and 68 %, respectively [23]. 

Deoxynivalenol, another member of the trichothecene mycotoxins, was purified from rice and the moldy corn culture of *F. graminearum* via high-speed counter-current chromatography, which is a hydrodynamic type of the liquid–liquid chromatography. This method produced 116 mg and 65 mg deoxynivalenol with a purity of greater than 94.9% from 200 g of the rice culture and the moldy corn, respectively. At these yields, the recovery rate of deoxynivalenol was 88% for both the rice culture and moldy corn based on the original deoxynivalenol concentration in the crude extracts analyzed by HPLC [24]. Similarly, a high-speed counter-current chromatography was used to purify the mycotoxin patulin produced by *Penicillium expansum* in sterilized apple juice. In that case, 21.9 mg of patulin was yielded from 50 mL of the apple juice culture while the purity and the recovery were 98.6% and 86.2%, respectively [25]. 

The purities of the AFs presented in our study fell into the range of 96.0 %–99.5%, which is comparable with the other purities achieved via the liquid–liquid chromatography which ranged from 77% to 103% for the reported mycotoxins, including deoxynivalenol, fumonisin B1-B3, fusarenon-X, nivalenol, and patulin. Furthermore, the recoveries were obtained in the range of 53.0%–90.0% and 69.7%–85.3% for the 250 mL and 1000 mL columns, respectively. These recoveries were higher on both columns than it was obtained at nivalenol (44%), and the maximum recoveries were higher than the recoveries of fumonisins B1 (68%), fusarenon-X (68%), patulin (86.2%), and deoxynivalenol (88%) presented in the literature.

## 4. Conclusions

In this study, a comprehensively optimized scale-up procedure of the CPC separation that aimed to purify AFs was successfully achieved. The maximum injection concentration of the crude material and the maximum injection volumes were determined on both a 250 mL and 1000 mL CPC column. With the application of the 250 mL rotor, altogether approximately 2.2 g of AFs could be purified with 8.2 liters of solvent daily (assuming an 8-hour workday), while on a 1000 mL column, approximately 7.8 g AFs could be prepared, utilizing around 31 liters of solvents per day. 

Based on our results, the most efficient CPC separation for the AFs purification can be achieved with the 1000 mL column injecting 100 ml of the crude extract via a pump injection regarding the purity and yield results. 

## 5. Materials and Methods

### 5.1. Chemicals and Solvents

All solvents used for preparative scale separations were analytical grade and were purchased from Molar Chemicals (Halásztelek, Hungary). The solvents used for HPLC-UV measurements were at least gradient grade or higher and were purchased from VWR International (Debrecen, Hungary). 

### 5.2. Preparation of the Crude Extract

AFs were extracted in four steps from the fermentation material of *A. parasicitus* SZMC 2473 strain (CBS 260.67; GenBank Accession number for ITS: MG662400), which is the ex-type of this species and originally isolated in Japan [26]. Cultivation of the fungus was carried out according to our previously described method [13].

For the preparation of the crude extracts, one liter of ferment broth was partitioned sequentially with 500 mL and 250 mL dichloromethane. The organic phases were combined and evaporated to water. Then, methanol and hexane were added to this aqueous residue, resulting in 45:50:120 volumetric ratio for aquatic sample/methanol/hexane ratio, respectively. After the phase separation, the upper phase was removed, and further extraction was performed on that phase with water/methanol (45:50 by volume). The separated water/methanol phases were then combined, and dichloromethane was added until two phases formed and extracted in two repetitions. The combined organic phases were dried over MgSO_4_, membrane filtered, and evaporated to dryness. From 4.5 L of fermentation material, approximately 1.2 g of crude material can be achieved. For all of the injections in this article, cultivation was carried out 7 times.

### 5.3. Centrifugal partition chromatography

Liquid–liquid separations were carried out on a 250 mL and a 1000 mL laboratory-scale CPC column built in same equipment unit (SCPC-250/1000, Gilson, Saint-Ave, France). The smaller volume rotor has a maximum rotation speed of 3000 rpm, creating a maximum of 725× *g* centrifugal force field, while the 1 L column has a maximum rotation speed of 1500 rpm, which equals to 515× *g* force field in the extraction cells. Rotors were coupled with a PLC250 flash/prep hybrid instrument (Gilson, Saint-Ave, France) containing a UV/VIS detector, fraction collector, electronically actuated injector valve with a 10 mL sample loop, an electronically actuated four-way two-position ascendant/descendant valve, and a manually actuated two-way two-position column selector valve. To control this instrument and acquire data, Gilson Glider Prep (Ver. 5.1) software (Gilson, Saint-Ave, France) was used.

For the separation of AFs, solvent systems were prepared according to our previous work [13]. At the beginning of the separation on both columns, the rotor speed was set to 500 rpm, representing 121× *g* and 172× *g* on the 250 mL and 1000 mL columns, respectively. In the case of the smaller capacity rotor, the separations were completely automatized. The stationary phase was pumped through the system for six minutes with 50 mL/min, then the flow rate was maintained 15 mL/min for the equilibration and separation, and the rotation speed was set to 1800 rpm equivalent to 435× *g*. The column was filled up with the stationary phase at 50 mL/min for 6 min before each injection. After the column was filled with the stationary phase, the mobile phase was pumped through to reach the hydrodynamic equilibrium. The equilibration and elution times were 20 min and 75 min, respectively, and the whole procedure lasted for 101 min. All separations were carried out in ascendant mode. During the separation, 20 mL fractions were collected. The 1 L rotor was filled up for 12 min with 100 mL/min with the stationary phase, then the flow rate was set to 60 mL/min during the equilibration and elution, while the rotor speed was held at 1267 rpm, achieving the same equivalent centrifugal field as on the smaller column. The equilibration and separations also lasted for 20 and 75 min, respectively, and 20 mL fractions were collected as well. UV detector was set to 366 nm during all separations.

### 5.4. HPLC-UV Analyses

Collected fractions were analyzed by HPLC-UV technique according to Endre et al. [13]. One milliliter aliquot of each collected fraction was evaporated and resolved in 500µL acetic acid. Then, 5 µL of this solution was injected onto the HPLC system (Shimadzu, Kyoto, Japan) equipped with a DGU-14A degasser, an LC-20AD binary pump, an SIL-20A autosampler, a CTO-10ASvp column thermostat, an SPD-10Avp UV-VIS detector, and a CBM-20A system controller. The data were acquired and evaluated with Class VP ver. 6.2 software (Shimadzu, Kyoto, Japan). For the stationary phase, a Gemini C18, 250 mm × 4.6 mm, 5 µm particle size column was applied (Phenomenex, CA, USA), while the isocratic mobile phase was the mixture of water/methanol/acetonitrile (60:20:20, v,v,v%). During the 16 min separation, the flow rate was held 1 mL/min and the column temperature was maintained at 40 °C. Peaks were detected at λ = 366 nm.

### 5.5. Final clean-up of AFs

Within a chromatographic separation the fractions containing the same single AF molecule (≥95%) were combined and their pH was neutralized with saturated NaHCO_3_ solution. The organic phases were dried over CaCl_2_, then were filtered, and evaporated to dryness. The dried AFs were kept under argon in the dark at −20 °C until HPLC-UV-HRMS measurements.

### 5.6. HPLC-HRMS analyses

Product purity was determined by HPLC-UV-HRMS technique using a Dionex Ultimate 3000 UHPLC system (Thermo Fischer Scientific, Waltham, MA, USA) equipped with a Dionex Ultimate Multi Wavelength UV-VIS detector and coupled with a Q-Exactive Focus Orbitrap mass spectrometer (Thermo Fischer Scientific, Waltham, MA, USA). Separation was carried out on a Phenomenex Gemini NX-C18 column (50 × 2.1 mm, 3µm) utilizing a H_2_O/MeCN gradient. The gradient program was the following: 0 min—5 B%, 1 min—5 B%, 10 min—95 B%, 11 min—95 B%, 11.2 min—5 B%, 15 min—5B%. UV data were collected at λ_1_ = 254 nm and λ_2_ = 366 nm. Product purity was determined at λ1 = 254 nm. The HRMS settings were as described in our previous work [13].

## Figures and Tables

**Table 1:** Yields and purities of the separations.

Column (mL)	Injected Amount		AFB_1_ (mg)	AFB_2_ (mg)	AFG_1_ (mg)	AFG_2_ (mg)	Total AFs (mg)	Recovery (%)^b^	Solvent Consumption (L)
**250**	90 mg (loop)	Yield (mg) Purity^a^ (%)	24 98.2	2 96.3	49 98.1	6 97.0	81 97.3	90.0	1.7
	180 mg (loop)	Yield (mg) Purity^a^ (%)	50 97.8	6 96.0	93 98.5	10 98.0	159 97.6	88.3	1.7
	210 mg (loop)	Yield (mg) Purity^a^ (%)	61 97.8	6 97.0	112 99.3	13 98.0	192 98.0	91.4	1.7
	240 mg (loop)	Yield (mg) Purity^a^ (%)	67 98.0	7 97.9	127 98.6	15 97.8	216 98.0	90.0	1.7
	250 mg (loop)	Yield (mg) Purity^a^ (%)	70 98.8	7 98.1	131 99.3	16 98.0	224 98.5	89.6	1.7
	250 mg (pump)	Yield (mg) Purity^a^ (%)	60 98.1	4 97.0	110 99.0	5 98.0	179 98.0	71.6	1.7
	375 mg (pump)	Yield (mg) Purity^a^ (%)	66 98.5	3 96.5	125 99.0	5 97.0	199 97.8	53.0	1.725
	500 mg (pump)	Yield (mg) Purity^a^ (%)	107 98.7	16 97.0	145 99.5	22 99.0	290 98.5	58.0	1.725
	625 mg (pump)	Yield (mg) Purity^a^ (%)	169 99.0	28 97.8	213 98.9	53 96.5	463 98.1	74.1	1.725
**1000**	1 g (pump)	Yield (mg) Purity^a^ (%)	320 98.3	29 96.9	434 99.0	70 99.0	853 98.3	85.3	6.9
	1.5 g (pump)	Yield (mg) Purity^a^ (%)	420 98.9	45 98.0	502 98.9	100 97.3	1067 98.2	71.3	6.9
	2 g (pump)	Yield (mg) Purity^a^ (%)	513 97.9	72 98.7	625 98.9	194 99.0	1404 98,6	70.2	6.9
	2.5 g (pump)	Yield (mg) Purity^a^ (%)	602 99.0	101 99.0	840 98.5	199 98.7	1742 98.8	69.7	6.9

^a^ Purities were calculated from the injections of each compound to HPLC-UV-HRMS. ^b^ Recoveries are calculated from each injected amount of crude extract in each run.

## Data Availability

We have full control of all primary data, and we agree to allow the journal to review our data if requested.
